# Sleep Duration, Quality, and Factors Affecting Sleep Health Among Young People in the California Bay Area: A Cross-Sectional Study

**DOI:** 10.7759/cureus.98394

**Published:** 2025-12-03

**Authors:** Ethan Y Pan, Sophia Li, Selina Li, Jasmine Ye, Benjamin Li, Austin Li, Christiane K Helmer, Joshua Ye, Nina W Helmer

**Affiliations:** 1 Public Health, Youth Sleep Initiative, Saratoga, USA; 2 Radiology and Biomedical Imaging, University of California San Francisco, San Francisco, USA; 3 Pediatrics, University of California San Francisco, San Francisco, USA; 4 Computer Science, Vanderbilt University School of Engineering, Nashville, USA; 5 Psychiatry, Greater Southern Alameda Area Kaiser Permanente, San Leandro, USA

**Keywords:** academic workload, adolescent health, adolescent sleep, sleep duration, sleep quality

## Abstract

Adequate sleep is essential for adolescent health, learning, and emotional well-being. However, youth sleep deprivation is a long-term growing public health challenge. This study investigates sleep duration and quality, factors significantly affecting sleep, perceptions, and resource awareness among young people in California’s San Francisco Bay Area. A cross-sectional survey was conducted between November 2024 and February 2025, involving 1,189 participants ranging from 10 to 24 years of age (in accordance with the WHO definition of young people), administered both in person and online. The instrument, reviewed by physicians for validity, assessed sleep duration, frequency of sleep disturbances, multiple sleep-affecting factors, students’ academic performance, emotional and behavioral challenges, perceived sleep needs, attitudes toward sleep, and awareness of resources. Descriptive statistical analyses and prevalence ratio analyses were conducted. The median sleep duration was 7 hours (mean = 6.68), which fell short of the recommended duration. While 1093 (91.9%) rated sleep as important, only 472 (39.7%) reported awareness of the available resources. Youth sleeping fewer than 8 hours faced a 1.80-fold higher likelihood of emotional or behavioral challenges compared with those meeting sleep recommendations (95% CI, 1.33-2.44; p < 0.05). Academic pressure emerged as the leading self-reported factor of sleep restriction, as our study revealed. Most Bay Area youth are not getting the sleep they need, even though they recognize its importance. However, limited awareness of resources reveals a critical gap. Findings highlight the need for community- and school-based sleep initiatives.

## Introduction

Adequate sleep during youth and early adulthood is crucial for physical, cognitive, and emotional development [1-3]. Adequate sleep is defined by the American Academy of Sleep Medicine as 8-10 hours of sleep for children 13-18 years of age [4], and the National Sleep Foundation (NSF) recommended 7-9 hours of sleep for adults 18-64 years of age [5]. Physically, sleep plays a vital role in the release of growth hormones necessary for proper bone and muscle development [6]. Additionally, adequate sleep helps regulate metabolism and supports the immune system, contributing to overall physical health [7]. Cognitively, sleep is fundamental for memory consolidation, learning, and problem-solving [8,9]. During sleep, the brain processes and organizes information acquired throughout the day, enhancing retention and facilitating the formation of new neural connections. Emotionally, sufficient sleep is crucial for mood regulation, stress management, and emotional resilience [3,10]. Adolescents who consistently get enough sleep are better equipped to handle the emotional challenges and stressors that come with this developmental stage, leading to improved mental health outcomes, academic performance, and overall well-being [11]. On the other hand, sleep deprivation in adolescents, characterized by inadequate sleep duration or quality, can lead to cognitive and social impairments, decreased academic performance, and an increased risk of mental health issues like depression, anxiety, stress, and suicidal ideation [12-14]. It also has negative effects on physical health, including a higher risk for obesity and hypertension [15-17].

Although numerous studies have explored sleep patterns in adults, there is a notable gap in comprehensive, large-scale surveys specifically targeting youth and young adult populations. One of the few available surveys was the Centers for Disease Control and Prevention’s (CDC) Youth Risk Behavior Survey (YRBS), which involved 9th through 12th grade high school students from all 50 states and the District of Columbia [18]. The survey primarily concentrated on sleep duration and revealed that in 2023, only 23% of high school students achieved at least 8 hours of sleep on an average school night. Furthermore, the proportion of both female and male students obtaining at least 8 hours of sleep on an average school night declined from 2013 to 2023, with male students decreasing from 35% to 25% and female students from 29% to 22%. Another annual nationwide sleep survey, the Sleep in America Poll, has been carried out by the NSF since 1991, but only covered the population aged 18 and above except for the years 2006 and 2024 [5]. Although sleep surveys targeting the youth and adolescent populations have been conducted outside of the United States (U.S.) [19-21], there is a lack of information on youth and young adults in the U.S. The lack of real-world survey data on sleep duration, sleep quality, factors affecting sleep health, and awareness of sleep-related resources among young people poses significant challenges [22]. These challenges make it difficult for researchers and policymakers to effectively address sleep-related issues in this demographic. This lack of data also hinders our understanding of the complex factors influencing sleep behaviors among young people, including the impact of technology use, academic pressure, caffeinated drinks, and social dynamics [23-25]. Furthermore, the scarcity of information on young people’s attitudes towards sleep and their awareness of available resources makes it difficult to develop targeted interventions and educational programs. Dysfunctional beliefs and intrusive thoughts about sleep have been shown to be related to insomnia and stress-induced sleep disturbances [26,27]. Efforts to enhance sleep health may not achieve their intended objectives without a comprehensive understanding of young people's perceptions and beliefs regarding the significance of sleep and the support they require.

This research aims to address critical gaps in understanding young people's sleep patterns, various sleep-affecting factors, and perceptions about sleep across different educational stages in the California Bay Area. The primary objective of this study was to assess actual sleep duration and sleep quality among young people in the California Bay Area. Secondary objectives were to identify factors associated with insufficient or disturbed sleep, such as academic workload, extracurricular commitments, electronic device use, and emotional or behavioral challenges; examine students’ perceptions of the importance of sleep and their ideal sleep duration; study the perceived impacts on academic performance, including concentration, memory, and grades; and evaluate awareness of available resources or strategies to improve sleep health. By identifying the risk factors and adverse consequences associated with insufficient sleep, this study seeks to inform targeted interventions and policies. This comprehensive approach will contribute to a more nuanced understanding of sleep disturbances among young people, potentially leading to more effective strategies to promote sleep hygiene and enhance overall well-being among young people across various educational levels.

## Materials and methods

Participants

Participants were eligible for inclusion if they were between 10 and 24 years of age, fitting the age group classified as young people by the World Health Organization (WHO) [28], and were residing in the California Bay Area at the time of survey completion. Participants were excluded if they fell outside the specified age range, did not reside in the Bay Area, or submitted surveys with illegible handwriting, incomplete answers, or missing essential data. Of the 1,226 survey responses collected, 37 were excluded, and 1,189 individuals between the ages of 10 and 24, with an average age of 17.6 years, were included. The study participants consisted of diverse gender and racial compositions and included students enrolled in both public and private schools. Educational levels included middle school, high school, college or university, and postgraduate studies. Geographically, participants were all from the Bay Area of Northern California, across 24 zip codes.

Survey instrument

This study utilized a cross-sectional survey targeting young people in the California Bay Area, primarily in high school and college or university. The survey was developed with reference to the Sleep Disturbance Scale for Children (SDSC), which was validated in adolescence [29], and in consultation with an American board-certified and licensed pediatric psychiatrist, C.Y., who specializes in adolescent health, reviewed the survey for validity. The study used convenience and volunteer sampling. The survey collected sociodemographic information and explored various aspects of sleep health, including sleep duration, sleep quality, potential risk factors affecting sleep, and the perceived impact of sleep on academic performance and emotional/behavioral state. Questions covered topics such as typical sleep hours during school nights, frequency of sleep disturbances, including trouble falling asleep or staying asleep at night or waking up too early in the morning, irregular sleep schedules, and sleep environment. Questions also addressed academic workload, extracurricular activities, electronic device usage before bed, family stress or changes, medical conditions/medications, heavy meals close to bedtime, lack of physical activity, time management issues, and social, emotional, and behavioral challenges. In addition, substance use was explored, including smoking/tobacco products, drinking alcohol, recreational drugs (cannabis, cocaine, ecstasy, etc.), and caffeine. The survey also gauged participants' opinions on the ideal sleep duration for young people and the importance of sleep. The design included both quantitative measures, such as hours of sleep, and qualitative elements, such as open-ended and multiple-choice questions about sleep health. This approach allowed for a comprehensive assessment of sleep patterns, behaviors, and perceptions among the target population. A copy of the survey form, including all survey questions in PDF format, is included in the supplement materials.

Data collection procedures

The survey was administered both online and in person using convenience and volunteer sampling. The online version was created using Google Forms and distributed through school newsletters at local high schools in the California Bay Area; participants were offered entry into a raffle for three $25 gift cards as an incentive. The in-person survey was printed in a single-page, double-sided format and distributed at multiple local public libraries as well as community college and university campuses. Informed assent and/or consent were obtained from all participants, and the completed paper surveys were subsequently digitized. The survey was anonymous, posed minimal risk, and focused on general sleep habits. Parental consent was not required; minors provided informed assent prior to participation, and all participation was voluntary. To prevent duplicate responses, the online survey was configured to allow only one submission per account. For in-person data collection, surveys were distributed and completed on-site, and each paper form was manually checked during data entry to ensure no duplicated handwriting, identical response patterns, or repeated demographic profiles were present. Anonymity and confidentiality were strictly maintained throughout data collection. The survey did not request any personally identifying information (e.g., name, contact information, student ID). The online survey did not record any names, emails, or IP addresses. Paper surveys were completed anonymously, stored securely, and digitized without any identifying information. All data were aggregated for analysis, and only summary-level results are reported.

Statistical analysis

Data were analyzed using descriptive statistics (means, medians, and percentages) in Microsoft Excel for Mac version 16.100.2. Bar graphs were used to show sleep hours reported by participants and the perceived ideal sleep duration. Tables were constructed to summarize the sociodemographics, sleep patterns, perceptions, and resource awareness. Prevalence ratio (PR) was calculated to assess the association between sleep adequacy (adequate vs. inadequate) and the presence of emotional or behavioral challenges. All p-values were two-sided, and the significance level was set at p < 0.05.

## Results

Sociodemographics

The study included 1189 participants aged 10-24 years (mean age 17.6 years) from the California Bay Area. Sociodemographic information is summarized in Table 1. The sample comprised 647 (54.4%) females, 512 (43.1%) males, 22 (1.9%) who preferred not to disclose, and 8 (0.7%) identifying as non-binary. The racial demographics were as follows: 737 (62.0%) Asian, 195 (16.4%) White, 91 (7.7%) multiracial, 21 (1.8%) Black or African American, 35 (2.9%) who preferred not to disclose, and 110 (9.2%) other racial groups. The majority of participants, 1100 (92.5%), were enrolled in public schools, while 89 (7.5%) attended private schools. The educational levels of the participants ranged from middle school to post-college graduates, with most participants in high school (621, 52.2%) and college or university (506, 42.6%). Geographically, all participants resided in the Bay Area of Northern California, representing 24 zip codes primarily located in the South Bay, including communities such as Los Gatos, Cupertino, and Saratoga.

**Table 1:** Sociodemographic characteristics of California Bay Area young people (N = 1,189).

Sociodemographic Characteristic	Mean (SD), Range /n (%)
Age (y)	17.6 (2.58), 10-24
Gender	
Male	512 (43.1%)
Female	647 (54.4%)
Non-binary	8 (0.7%)
Prefer not to specify	22 (1.9%)
Race/Ethnicity	
Asian	737 (62.0%)
White	195 (16.4%)
Multiracial	91 (7.7%)
Black/African American	21 (1.8%)
Middle Eastern or Northern African	27 (2.3%)
Native Hawaiian or other Pacific Islander	4 (0.3%)
Native American or Alaskan Native	3 (0.3%)
Other	76 (6.4%)
Prefer not to specify	35 (2.9%)
Education level	
Middle school	56 (4.7%)
High school	621 (52.2%)
College/University	506 (42.6%)
Postgraduate	6 (0.5%)

Sleep duration and quality

**Figure 1:**
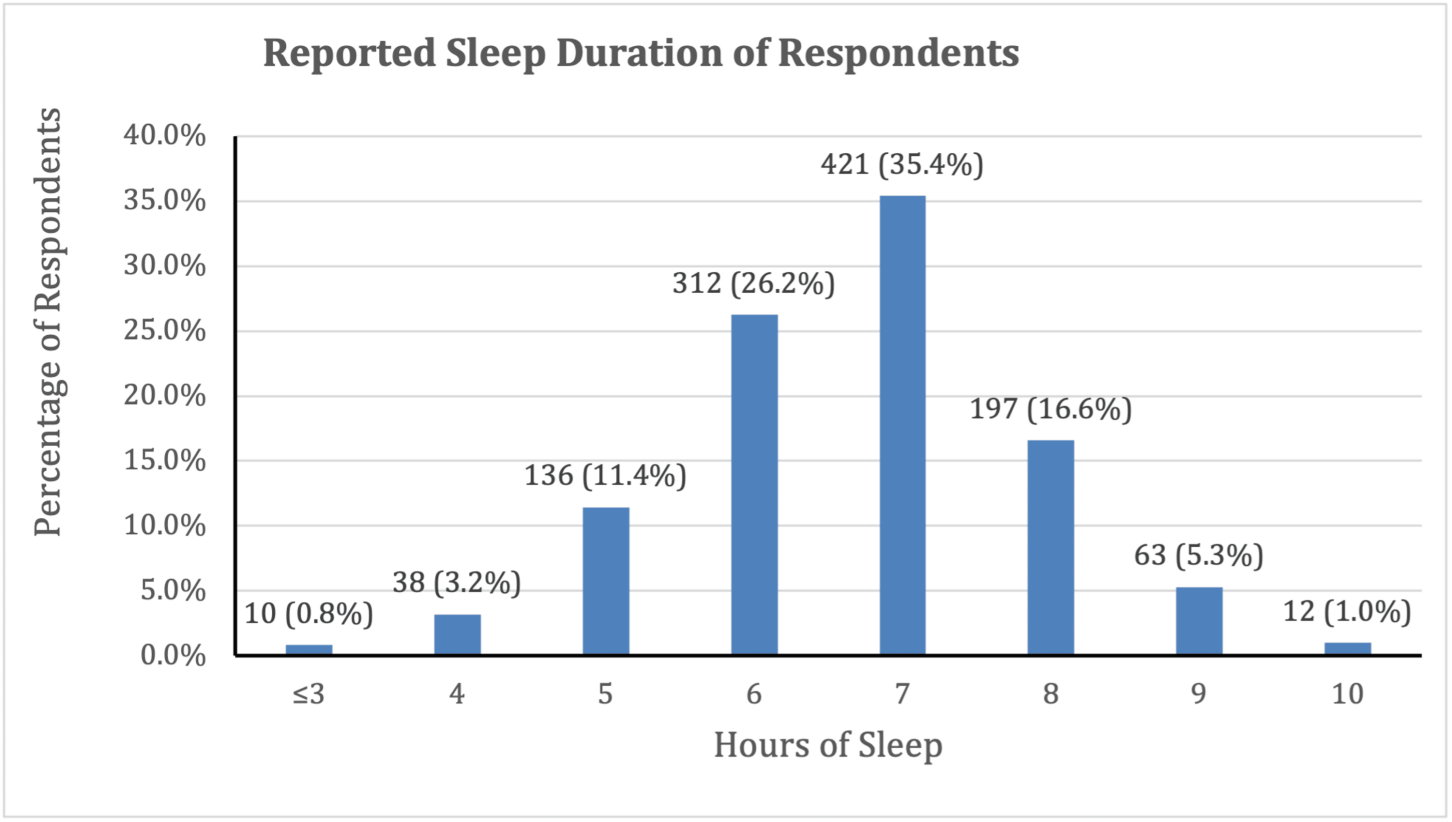
Reported hours of sleep during school nights (N=1,189).

The overall median reported sleep on school nights was 7 hours, and the mean sleep duration was 6.68 hours, with a wide range of sleep durations from as few as <3 hours to as much as 10 hours (Figure 1). High school and college students and post-college graduates reported median values of 7 hours, and middle school students reported a median sleep duration of 8 hours. The mean sleep duration was 8.30 hours for middle school students, 6.68 hours for high school students, 6.52 hours for college/university students, and 6.60 hours for post-college graduates. School night sleep duration by educational stage was summarized in Table 2, which highlights the decline in average sleep hours with advancing educational levels.

**Table 2:** School night sleep duration by current education level.

Education Level	n (%)	Mean Sleep (hrs.)	Median Sleep (hrs.)	Number of Days Sleep < 8 hrs.
Middle school	56 (4.7%)	8.30	8	1.89
High school	621 (52.2%)	6.68	7	3.58
College/University	506 (42.6%)	6.52	7	3.50
Postgraduate	6 (0.5%)	6.60	7	3.6
Overall	1189 (100%)	6.68	7	3.32

A significant number of students reported days on which they slept less than 8 hours, with 420 (35.3%) students sleeping less than 8 hours all five days of a school week, 275 (23.1%) students reporting four days a week, and 171 (14.4%) students sleeping less than three days. There was a trend of older students reporting slightly more days on which they slept less than 8 hours (e.g., a mean of 3.52 for college/university students vs. 3.32 for high school students).

The frequency of sleep disturbances, which included trouble falling asleep or staying asleep at night or waking up too early in the morning, is summarized in Table 3. A total of 444 (37.3%) of the participants reported that they rarely experienced these sleep disturbances. Of the participants, 410 (34.5%) reported sleep disturbances 2-3 nights per week, and 262 (22.0%) reported having sleep disturbances 4-6 nights per week. A total of 73 (6.1%) of the participants reported always having sleep disturbances.

**Table 3:** Reported frequency of sleep disturbances.

Frequency of Sleep Disturbances	n (%)
Rarely (0-1 night)	444 (37.3%)
Sometimes (2-3 nights)	410 (34.5%)
Often (4-6 nights)	262 (22.0%)
Always (7 nights)	73 (6.1%)
Overall	1189 (100%)

Factors affecting sleep and functional status

Various factors have been reported to negatively affect sleep quality and quantity in participants (Table 4). Academic workload was reported to significantly affect sleep by 971 (81.8%) of participants. This was followed by electronic device use (709, 59.7%), time management issues (659, 55.5%), extracurricular activities (646, 54.4%), irregular sleep schedule (551, 46.4%), and emotional challenges, including personal stress and anxiety (504, 42.5%).

**Table 4:** Participant-reported academic, behavioral, lifestyle, and other factors potentially affecting sleep.

Category	n (%)
Academic Workload	971 (81.8%)
Extracurricular Activities	646 (54.4%)
Family Stress or Changes	415 (35.0%)
Social Challenges (e.g., bullying or being bullied)	84 (7.1%)
Use of Electronic Devices before Bed	709 (59.7%)
Emotional Challenges (e.g., personal stress, anxiety)	504 (42.5%)
Behavioral Challenges (e.g., ADHD)	138 (11.6%)
Medications	56 (4.7%)
Smoking/Tobacco Products	34 (2.9%)
Drinking Alcohol	28 (2.4%)
Recreational Drugs (e.g., cannabis, cocaine, ecstasy, etc.)	23 (1.9%)
Caffeine	179 (15.1%)
Sleep Environment (e.g., noise, temperature)	311 (26.2%)
Irregular Sleep Schedule	551 (46.4%)
Heavy Meals close to Bedtime	187 (15.8%)
Lack of Physical Activity	185 (15.6%)
Time Management Issues	659 (55.5%)

In terms of electronic device use, 376 (31.6%) students reported using their devices for more than 4 hours per day. A total of 392 (33.0%) students reported 2-4 hours of daily use. A total of 321 (27.0%) reported 1-2 hours per day, and 100 (8.4%) participants reported less than 1 hour a day.

On a scale of 1-5, with 5 being the most significant effect and 1 being no effect, the participants rated the effect of academic workload on sleep. A total of 323 (27.2%) students gave a score of “5,” 411 (34.6%) gave a score of “4,” 292 (24.6%) gave a score of “3,” 126 (10.6%) gave a score of “2,” and 37 (3.1%) gave a score of “1.”

In addition, the effect of extracurricular activities on sleep was rated semiquantitatively. A total of 569 (47.9%) participants thought extracurricular activities affected sleep, but only slightly. A total of 253 (21.3%) students believed that extracurricular activities significantly affected their sleep. Of the respondents, 258 (21.7%) did not think extracurricular activities had a significant effect on their sleep, and 109 (9.2%) reported that they did not participate in extracurricular activities.

Sleep and function/performance

When asked to rate the impact of their sleep duration on academic performance, including concentration, memory, and grades, on a scale from 1 (no effect) to 5 (significant effect), 280 participants (23.5%) selected “5,” 368 (31.0%) selected “4,” 340 (28.6%) selected “3,” 152 (12.8%) selected “2,” and 49 (4.1%) selected “1.”

A total of 278 (23.4%) participants reported emotional or behavioral challenges (e.g., anxiety, ADHD, depression) that interfered with their sleep. Prevalence ratio analysis was performed with the exclusion of those who responded “prefer not to say.” Participants who slept fewer than 8 hours had a 1.8-fold higher likelihood of experiencing these challenges compared with those who slept at least 8 hours (95% CI, 1.33-2.44, p < 0.05) (Table 5).

**Table 5:** Prevalence ratio analysis of sleep duration and emotional/behavioral challenges.

	Yes	No
Sleep <8 hours	240	610
Sleep ≥8 hours	40	215
Prevalence ratio (95% CI)	1.80 (1.33 – 2.44)	

A significant number of young people (n = 383, 32.2%) answered yes when asked if they had ever driven while feeling sleepy or drowsy. This accounted for 54.0% of the students who were driving. A total of 480 (40.4%) students reported that they were not driving at the time of the survey.

Perception, awareness, and resources

**Figure 2:**
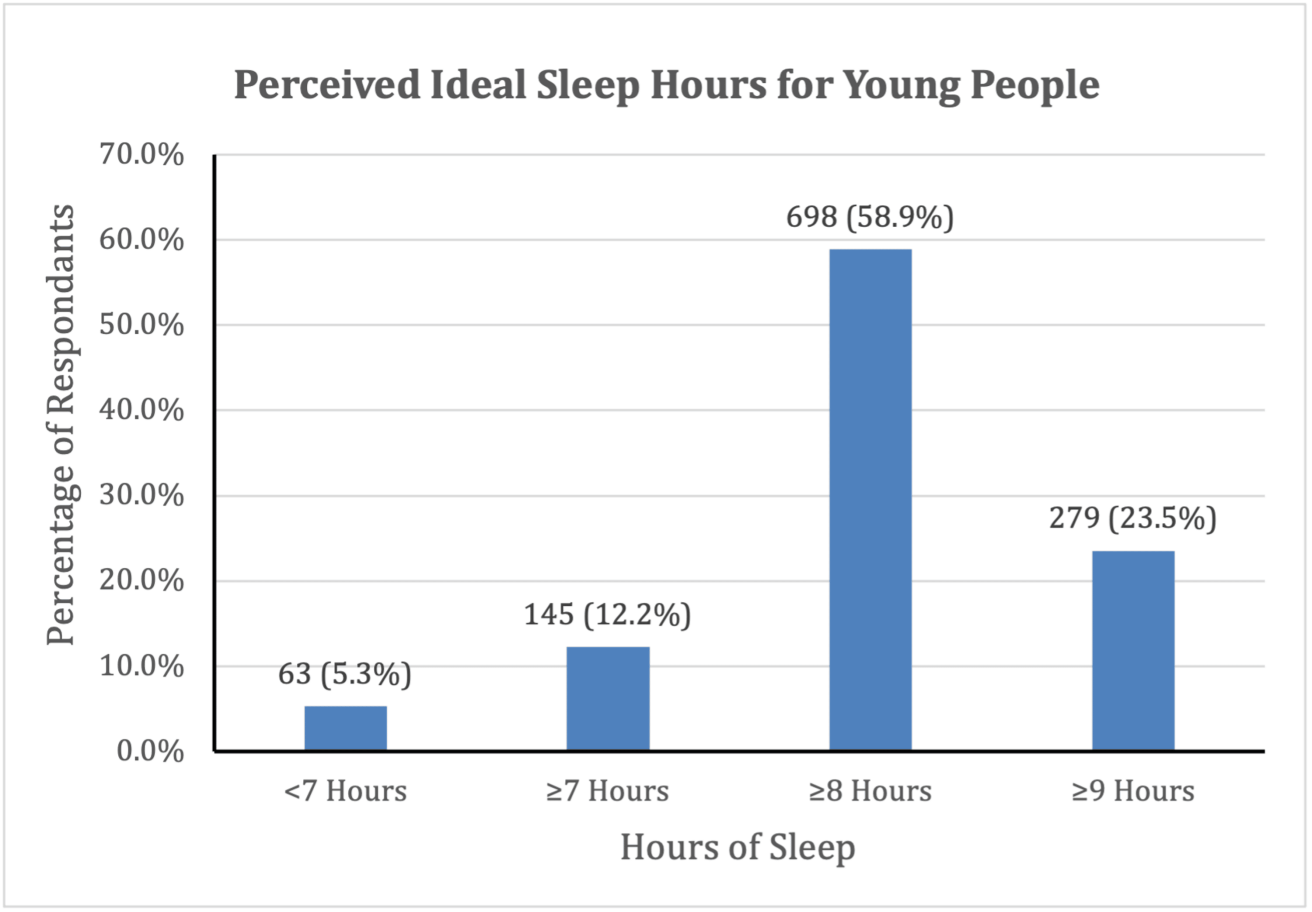
Perceived ideal sleep duration for young people (N=1,189).

Young people generally recognize the need for more sleep. A total of 700 (58.9%) young people reported 8 hours or more as the ideal sleep duration, and 279 (23.5%) reported at least 9 hours (Figure 2). The median perceived ideal sleep duration was 8 hours. Nearly all respondents, 1093 (91.9%), rated sleep as important, with scores of 4-5 on a 5-point scale. Despite recognizing the importance of sleep, only 472 (39.8%) of respondents reported awareness of resources or strategies to improve sleep.

## Discussion

Nationally and internationally based sleep surveys have revealed a significant discrepancy between the recommended and actual sleep duration among youth and a gradual decline in adolescent sleep over a 20-year period from 1991 to 2012 [2,5,17,18,30,31]. While the American Academy of Sleep Medicine recommends 8-10 hours of sleep for children 13-18 years of age [4] and the NSF recommends 7-9 hours of sleep for young adults [5], our study found that many fall short of this target. The overall median reported sleep duration on school nights was 7 hours, and the mean sleep duration was 6.68 hours, with a wide range of sleep duration from as few as 2-3 hours to as much as 10 hours. Our study revealed that sleep deprivation is highly prevalent, with 560 (71.0%) of youth aged 10-18 sleeping less than the recommended 8-10 hours and 362 (90.5%) of young adults aged 18-24 sleeping less than the recommended 7-9 hours, potentially impacting their overall well-being and academic performance. This is consistent with the CDC’s YRBS, which showed that during 2023, only 23% of high school students got at least 8 hours of sleep on an average school night [18], and the NSF’s Sleep in America Poll, which found that 8 of 10 teens did not get enough sleep [5]. There was a clear trend of decreasing sleep duration with age, with a mean sleep duration of 8.30 hours for middle school students, 6.68 hours for high school students, and 6.52 hours for college or university students. Our study also showed a high prevalence of young people experiencing sleep quality issues, such as trouble falling asleep, staying asleep at night, or waking up too early in the morning. In addition, slightly less than two-thirds of participants reported relatively frequent sleep complaints ranging from 2-3 days/week to seven days/week.

Among the factors contributing to insufficient sleep, academic workload was most frequently reported, with 971 (81.8%) of participants indicating that it significantly affected their sleep quality. More than half of the participants cited electronic device use, time management difficulties, and extracurricular activities as additional contributors. Other notable factors included irregular sleep schedules and emotional challenges, including personal stress and anxiety. Academic and social pressures are significant contributors to insufficient sleep in adolescents, particularly in high-achieving communities where academic excellence is prioritized [1,32,33]. The use of electronic devices, especially smartphones, tablets, and computers, plays a crucial role in sleep deprivation by disrupting natural sleep-wake cycles [34]. Notably, 376 (31.6%) of students in our study reported using electronic devices for more than four hours per day. In the context of the digital era, this finding is particularly concerning, as recent studies have demonstrated that excessive screen time is associated with a range of mental health symptoms, especially depressive symptoms [35], and that addictive patterns of screen use confer increased risk to adolescent mental health, including suicidality [36]. 

Our study participants generally recognized the importance of sleep for their health and performance, with 980 (82.4%) of them considering at least 8-9 hours of sleep per day to be ideal. However, their actual sleep duration fell significantly short of the perceived ideal sleep duration. This gap may be particularly pronounced in communities with high-pressure academic environments, such as those represented in the study (e.g., Los Gatos, Cupertino, and Saratoga). While students may understand that sleep is crucial, they may struggle to implement effective strategies for better sleep hygiene amid demanding schedules and academic expectations, and only 472 (39.8%) of participants were aware of resources to assist sleep and improve sleep health.

Research has consistently demonstrated the negative impact of sleep impairment on cognitive function, academic performance, and emotional well-being among youth and young adults [1-3,12,14,20,31,37-39]. Our study corroborates this notion and finds that insufficient sleep and poor sleep quality are associated with impaired academic performance, including concentration, memory, and grades. Additionally, our study showed that sleep deprivation is associated with an increased risk of behavioral and emotional challenges. These effects are particularly concerning for the study's participants, who are navigating critical developmental stages and facing increasing academic challenges in middle school, high school, and college/university. Our data showed that insufficient sleep may also pose a safety issue, with 383 participants, 32.2% of all participants, and 54.0% of participants who drove a car reporting that they had experienced drowsy driving.

Our findings align with national surveys highlighting chronic sleep deprivation among U.S. youth [2,5,18] and similarly reflect the compromised sleep duration and quality prevalent among local youth, an issue that warrants attention. The implications of insufficient sleep among adolescents are multifaceted, with academic and social demands emerging as key contributors. The pressure to excel academically, particularly in high-achieving communities where our study participants reside, often leads students to prioritize studying and homework over adequate sleep. This is compounded by increasing social expectations, electronic device use, and extracurricular activities that compete for teenagers' time and attention. Therefore, it is not surprising that time management issues were the second most reported factor affecting sleep, reported by 659 (55.5%) of the participants.

The study's limitations include the reliance on self-reported data, which may introduce recall bias, potentially affecting the accuracy of sleep duration and quality reports. Our analyses did not include multivariate adjustment for potential confounders such as age, gender, or educational level, and the survey instrument, although reviewed by clinicians for content validity, did not undergo external validation or formal psychometric testing, which may limit the robustness and generalizability of the findings. Our study may also be subject to selection bias due to the voluntary, online distribution of part of the survey, which may have preferentially attracted students with particular interest or concerns about sleep. In addition, all sleep measures were self-reported, potentially introducing response bias, and the lack of objective data (e.g., actigraphy or wearable sleep metrics) may limit the accuracy and precision of the sleep estimates. The cross-sectional design precludes the establishment of causality between the identified factors and sleep patterns. Future longitudinal or interventional studies may be warranted to confirm the associations identified in this study. The survey questions on sleep duration did not allow for smaller time increments (e.g., half-hour intervals), which may have reduced precision. The majority of our participants were recruited from public schools, which may introduce sampling skews and limit the socioeconomic representativeness of the sample. Another limitation is that the data collection procedure may introduce sampling bias, as participants were primarily recruited from specific schools, libraries, and college campuses, which may not fully represent the broader population. Similarly, given that the majority of our sample was drawn from the South Bay, these findings may not be generalizable to the broader California Bay Area or U.S. youth populations. Despite these constraints, the findings align with national surveys highlighting chronic sleep deprivation among U.S. youth.

## Conclusions

The study revealed that young people commonly sleep less than the recommended hours, and many have frequent sleep disturbances, significantly impacting their cognitive performance, including concentration, memory, academic grades, and emotional/behavioral well-being. Despite valuing sleep, most students lack access to effective resources. This aligns with national surveys highlighting chronic sleep deprivation among American youth. Academic and social pressures, particularly in high-achieving communities, contribute significantly to insufficient sleep among adolescents. The use of electronic devices, especially smartphones and computers, disrupts natural sleep-wake cycles. Time management emerges as a critical and manageable factor, with many adolescents struggling to balance commitments effectively. Our findings suggest that multifaceted strategies may help improve sleep health among young people. By focusing on these controllable aspects, it may be possible to mitigate the negative impact of insufficient sleep on adolescents' cognitive function.

## Figures and Tables

**Figure 3:**
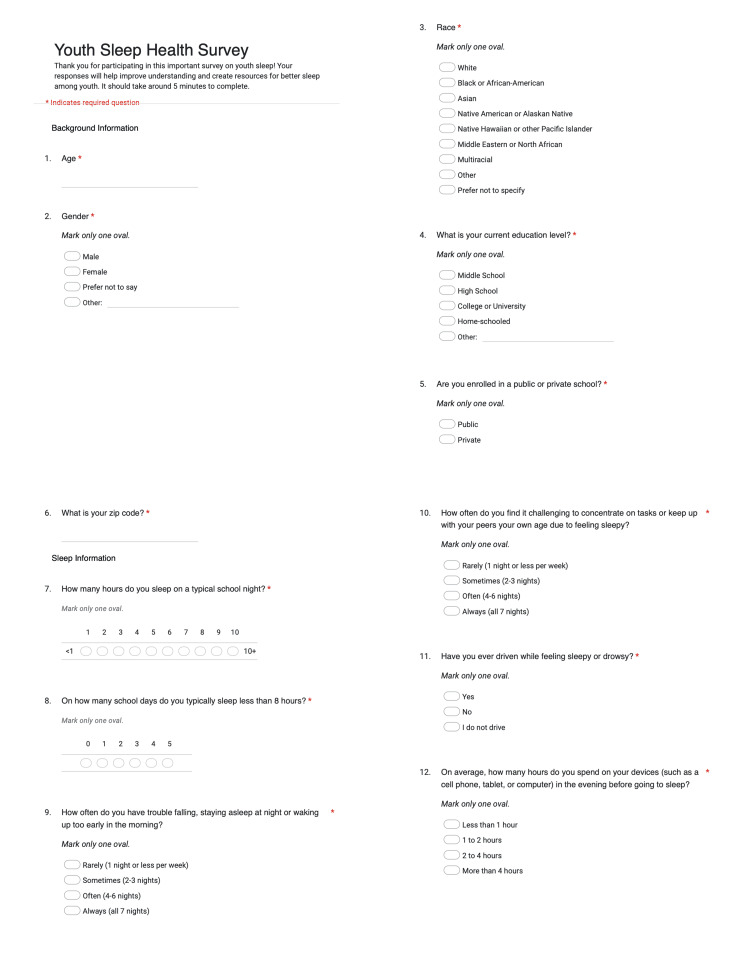
Page 1/2 of the survey form.

**Figure 4:**
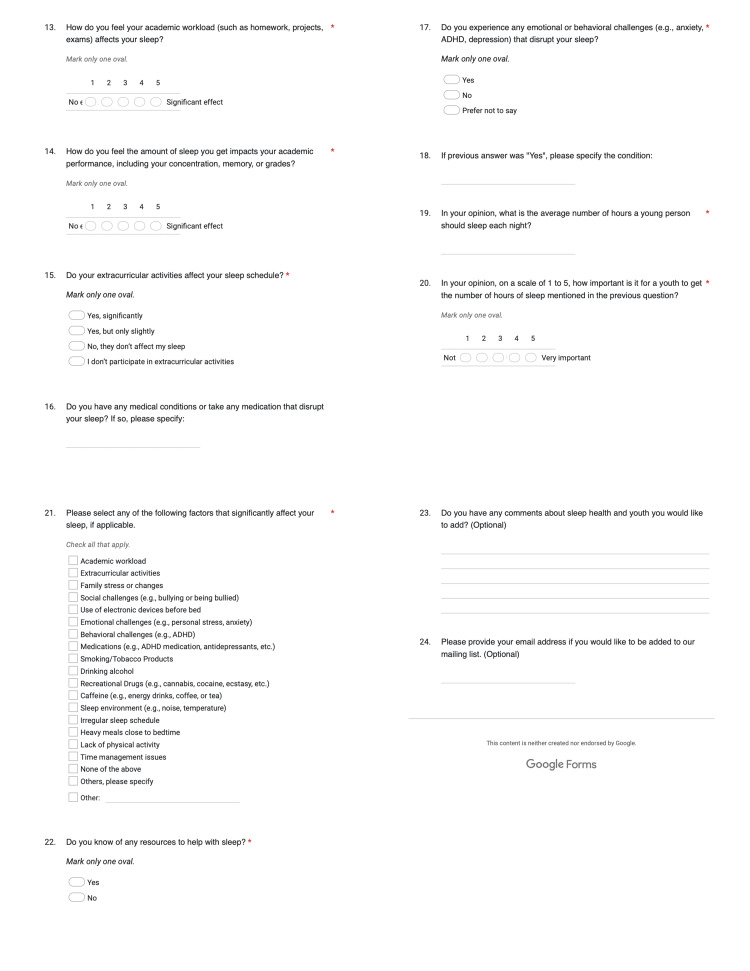
Page 2/2 of the survey form.
